# TabNet-driven interpretable prediction of multi-oxide composition in cement using NIR spectroscopy

**DOI:** 10.3389/fchem.2025.1691413

**Published:** 2025-10-30

**Authors:** GeZhiChen Li

**Affiliations:** School of Information Science and Engineering, Shandong Normal University, Jinan, China

**Keywords:** cement raw meal, near-infrared (NIR) spectroscopy, deep learning, oxides prediction, regression algorithm

## Abstract

**Introduction:**

Accurate monitoring of oxide compositions is critical for ensuring cement quality and performance in industrial production. Conventional analytical techniques for this purpose are often time-consuming, costly, and lack real-time capability. While Near-infrared (NIR) spectroscopy offers a rapid and non-destructive alternative, traditional chemometric models struggle to capture the highly nonlinear, high-dimensional spectral characteristics and exhibit limited interpretability.

**Methods:**

To address these challenges, this paper proposes an interpretable TabNet-based multi-output regression method for predicting multiple oxide concentrations from NIR spectra. The proposed method integrates sparse feature selection with adaptive information aggregation, enabling it to dynamically prioritize the most informative spectral regions during processing. This architecture facilitates both automatic wavelength selection and accurate oxide content prediction.

**Results:**

Extensive experiments on two cement datasets demonstrate that the proposed TabNet model consistently outperformed established baseline models in predictive accuracy. A key advantage of the TabNet framework is its enhanced interpretability, achieved by generating sequential attention masks that highlight chemically meaningful wavebands associated with each oxide component.

**Discussion:**

This framework provides a scalable and insightful solution for spectral-based analysis, not only for cement quality monitoring but also for other materials science applications. The code is available at https:// github.com/Andrew-Leopard/CementOxidePredictor.

## 1 Introduction

Cement is a cornerstone of the global construction industry, and its manufacturing quality largely depends on the precise control of raw meal composition (Sambataro et al., 2024; Yang et al., 2022). The concentrations of key oxides (SiO_2_, Al_2_O_3_, Fe_2_O_3_, CaCO_3_) play a decisive role in governing clinkering reactions, mineral formation, and overall product stability (Castillo et al., 2025). Rapid and accurate prediction of these oxides is essential for improving dosing accuracy, energy efficiency, and cement quality stability (Hu et al., 2025). Traditional oxide quantification techniques, such as X-ray fluorescence and wet chemical analysis, provide high accuracy but are time-consuming, labor-intensive, and costly, making them unsuitable for real-time quality control in large-scale cement production (Polavaram and Garg, 2021). Near-infrared (NIR) spectroscopy has emerged as a promising analytical tool for cement raw meal analysis because it is reagent-free, non-invasive, and capable of providing rapid measurements (Casson et al., 2020; Haruna et al., 2023). However, modeling the complex spectral-chemical relationship remains a challenge due to the high dimensionality and redundancy of NIR data, coupled with nonlinear interactions among variables.

Recent advances in machine learning (ML) have greatly improved the analysis of high-dimensional spectral data, enabling robust predictions of material properties (Huang et al., 2021; Wu et al., 2024). Such as partial least squares regression (PLSR) and Random Forest, have been widely used for NIR spectral modeling (Alessandra and Tormod, 2019; Mishra et al., 2021). A key limitation of these methods is their dependence on manual preprocessing and dimensionality reduction, which restricts their capacity to model the intricate nonlinearities in cement spectral data (Zhang Y et al., 2024). Hybrid approaches have attempted to improve predictive accuracy through feature-level spectral fusion and wavelength selection (Acquarelli et al., 2017; Ma et al., 2025). For example, Haruna et al. used NIR with multivariate calibration for rapid phenolic analysis in peanuts, and later integrated LIBS and NIRS to improve oxide quantification in cement (Haruna, et al., 2023). Despite these efforts, the existing methods remain reliant on handcrafted feature engineering and lack interpretability (Lee and Kim, 2016). Deep learning enables automatic feature extraction and relationship modeling, offering a promising solution for hyperspectral data analysis (Qu et al., 2021). However, most deep learning models require large amounts of training data, which are difficult to obtain in industrial cement production. At present, there is a lack of deep learning-based predictive frameworks specifically designed for oxide analysis in cement raw meal.

To tackle the above challenges, this paper propose a novel multi-output regression model inspired by the TabNet for accurate oxide content estimation (Arik and Pfister, 2021). TabNet is a deep learning architecture specifically designed for structured tabular data, combining the interpretability of decision trees with the representation power of neural networks. Compared with conventional deep learning approaches such as convolutional neural networks (CNNs) or recurrent neural networks (RNNs), which are typically designed for image or sequential data, TabNet offers several unique advantages that make it particularly suitable for NIR spectral analysis. Specifically, the method combines feature and attentive transformers with sparsemax activation and multiple decision steps, for effective modeling of nonlinear patterns in complex spectral data. The TabNet integrates feature learning, selection, and interpretation in a unified framework, making it particularly suitable for high-dimensional spectral data. This paper conduct a series of experiments to evaluate method performance, analyze feature attribution patterns, and explore inter-regional spectral dependencies. Extensive experiments on two real-world datasets from Qufu and Linyi demonstrate that TabNet significantly outperforms traditional models in both RMSE and R^2^ across all oxides. The method achieves an average R^2^ of 0.922 and RMSE of 0.097 on the Qufu dataset, and interpretable attention maps that align with known molecular vibration bands. In the future, this method can be broadly applied to other inorganic materials, geological analyses, and real-time industrial process monitoring. The main contributions of this work are as follows:

1. This paper develop a novel TabNet-based regression method that leverages shared representations to capture correlations among multiple oxides. This architecture achieves superior predictive accuracy while demonstrating remarkable generalization capability across different production regions, establishing a new paradigm for multi-output spectral analysis.
2. This paper develop an end-to-end analytical system that automatically processes raw high-dimensional NIR spectra without manual intervention. The framework seamlessly integrates wavelength selection and concentration prediction within a unified architecture, effectively eliminating the dependency on conventional feature engineering.
3. This paper establish an interpretable spectral-oxide correlation paradigm through a sparse attention mechanism to identify key spectral bands associated with oxide-specific molecular vibrations, providing intuitive interpretability of the spectral feature information.

## 2 Materials and methods

### 2.1 Overview

**FIGURE 1:**
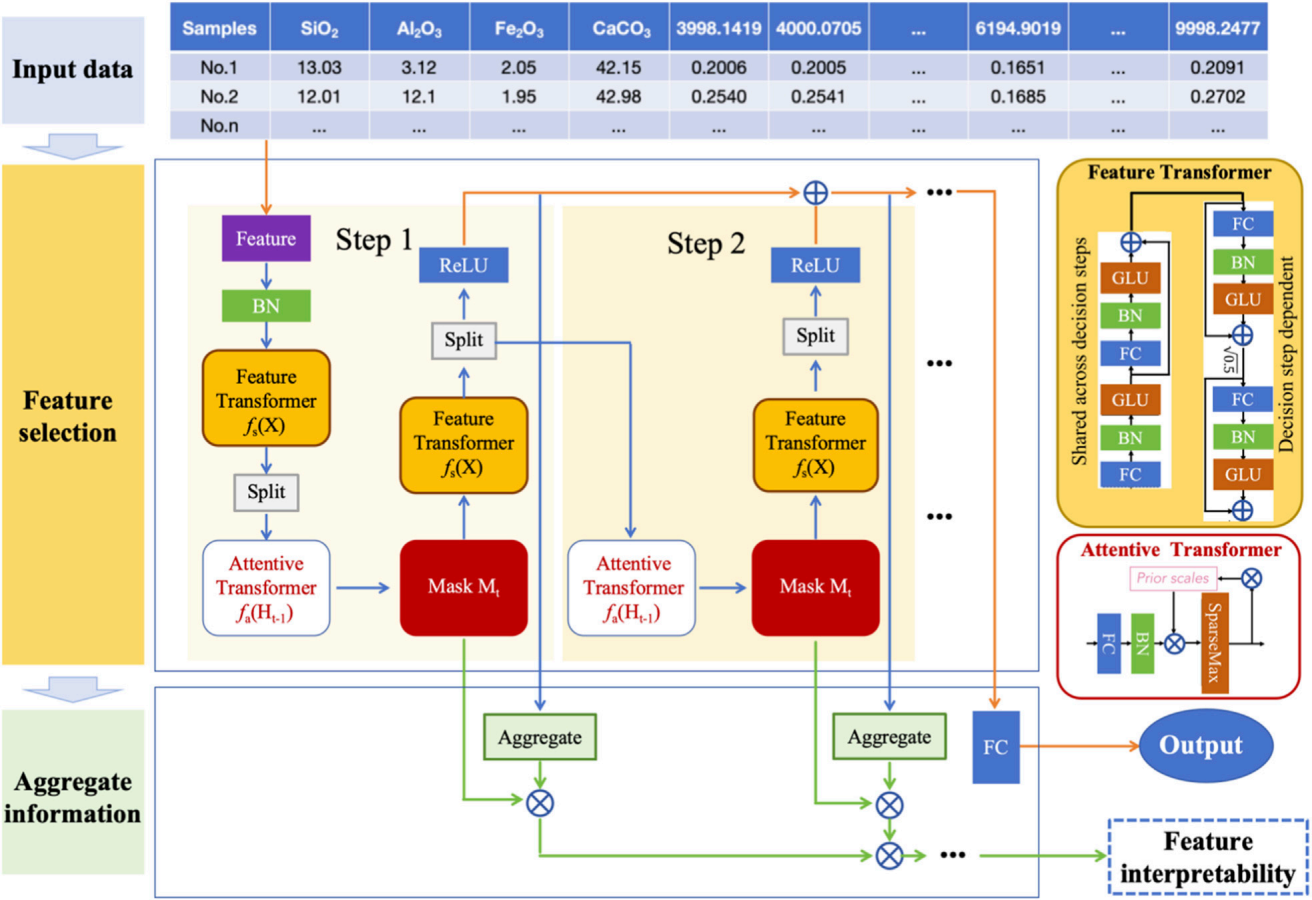
Overall architecture of the TabNet-based regression method for multi-oxide prediction.

Cement quality is critically determined by the concentration of major oxides in raw meal, including SiO_2_, Al_2_O_3_, Fe_2_O_3_, and CaCO_3_. To predict cement oxide contents from spectral data, this paper propose a TabNet-based regression method that integrates feature learning, selection, and interpretation within a unified framework. The overall architecture is shown in Figure 1, which mainly includes three parts: input data, feature selection and aggregation information. The model first transforms the input spectral data using a feature transformer and batch normalization. At each decision step, an attentive transformer generates sparse feature masks to dynamically select the most relevant wavelengths. These masked features are processed to produce partial predictions, which are aggregated across steps to form the final output.

Importantly, the accumulated attention masks enable direct interpretation of feature importance, linking spectral bands to specific oxides. This approach ensures both high accuracy and strong interpretability for multivariate spectral prediction tasks. The body of TabNet comprises a shared feature transformer, a decision step block with a unique attention mask, and a sparsemax function to generate interpretable feature selection masks. Its end-to-end differentiable architecture integrates both feature learning and prediction, eliminating the need for manual feature engineering. The following sections will provide a detailed introduction to each component of the proposed method and its corresponding function.

### 2.2 Method details

Given the high-dimensional NIR spectral data $X\in R^{n\times d}$ , where d represents the number of spectral wavelengths and n the number of samples. The input to the feature selection module consists of normalized spectral data, processed through standard preprocessing steps such as scaling and standardization. The goal is to predict the concentration values of four oxide components $Y\in R^{n\times 4}$ simultaneously. In contrast to single-object models, our approach adopts a multi-output regression framework to leverage interdependencies among the predicted oxides.

The feature selection module primarily comprises two components: the Feature Transformer and the Attentive Transformer. The model processes the input features through a sequence of decision steps $t\in \left\{1,...,T\right\}$ , starting with a shared Feature Transformer that applies nonlinear transformations to produce an embedding, denoted as $H_{0}=f_{s}\left(X\right)$ . This shared transformation is reused across all decision steps to reduce parameter redundancy and ensure consistent feature representation. At each t , TabNet maintains a complementary feature $H_{t}$ and latent feature $Z_{t}$ , which is updated as follows Equation 1:

$$H_{t}=\text{BatchNorm}\left({\;Z}_{t}+H_{t‐1}\right)=\text{BatchNorm}\left({\;f}_{s}\left(X_{t}\right)+H_{t-1}\right)$$ (1)

This mechanism forces the model to select only a few relevant spectral bands at each decision step. Then, the masked input is processed by a shared feature transformer to produce an intermediate decision vector $h_{t}\in H_{t}$ (Equation 2):

$$h_{t}=\text{Feature Transformer }\left(X_{t}\right)=\text{ReLU}\left(W_{t}\cdot X_{t}+b_{t}\right)$$ (2)

where, $W_{t}$ and $b_{t}$ are represent the weight and bias of function, respectively.

The detailed structure of the Feature Transformer module is illustrated in the yellow block on the right side of Figure 1. It consists of two components: a shared block across all decision steps and a decision step–dependent block. The shared block includes a sequence of batch normalization layers, linear projections, and gated linear units (GLUs), which collectively enable the model to capture complex nonlinear dependencies and shared feature representations throughout the decision step. The decision-dependent block, unique to each decision step, is designed to inject step-specific information into the learning process. It follows a similar architecture but operates independently across steps, allowing the network to dynamically refine representations based on the evolving attention masks and selected features at each step.

Then, the Attentive Transformer module receives the hidden representation $h_{t}$ from the previous step $H_{t-1}$ and generates a sparse feature selection mask $M_{t}\in {\left[0,1\right]}^{d}$ , which guides feature selection at the current step. This module comprises a fully connected (FC) layer, normalization, and a nonlinear activation function GLUs. The output of these layers is then element-wise multiplied with the prior scale vector, which tracks the selection probability of each feature over steps to prevent redundant reuse. This operation yields a weighted activation vector that is passed through a sparsemax activation function, which projects the values onto a sparse probability simplex (Equation 3):

$$M_{t}=\text{sparsemax}\left(f_{a}\left(H_{t-1}\right)\right)$$ (3)

where $f_{a}\left(.\right)$ is the attention transformation network. TabNet departs from traditional dense models by introducing adaptive sparsity into feature selection. To ensure interpretability and computational efficiency, TabNet applies a sparsemax activation to compute the attention mask. Unlike softmax, which produces smooth but dense probability distributions, sparsemax maps input activations to a sparse probability simplex. The sparsemax function ensures that only a subset of features is selected at each step: an attention mask is applied to select informative wavelengths. This mask is generated from a learnable attention module using a sparsemax activation (Equation 4):

$$\text{sparsemax}{\left(z\right)}_{i}=\max\left(0,z_{i}-\tau \right),\text{where }\tau \text{ solves }\sum_{i}\;\max\left(0,z_{i}-\tau \right)=1$$ (4)

The masked feature vector is computed via element-wise multiplication with the shared embedding (Equation 5):

$$X_{t}=M_{t}⊙H_{0}$$ (5)

Interpretability is embedded directly into TabNet’s architecture through the accumulation of attention masks over time. It generates a sequence of masks $\left\{M_{1},M_{2},\ldots ,M_{T}\right\}$ . These are combined to form a global feature importance measure, the Equation 6 is:

$$\text{Feature Importance}=\sum_{t=1}^{T}\;M_{t}$$ (6)

This summation represents how frequently each feature (i.e., wavelength) was selected across all steps. This provides a global feature attribution profile, enabling identification of key wavelengths relevant to prediction, both globally and per oxide.

Next, the multi-step aggregation information key is iteratively refined feature usage over decision steps. The masked input is then processed by a step-specific Feature Transformer to generate a latent representation. This representation is used to produce a partial prediction $P_{t}$ for the target variables. The module consists of a stack of fully connected layers (FC) with batch normalization (BN), ReLU activation, and ghost batch normalization for regularization. Simultaneously, a complementary stream preserves unselected information, allowing the model to revisit previously ignored features in future steps. $P_{t}=f_{p}\left(Z_{t}\right)$ that contribute to the model’s partial prediction. $f_{p}\left(.\right)$ function usually uses multiple FC layers to implement oxide prediction.

TabNet models prediction as a multi-step decision process, analogous to boosting or residual learning. Instead of producing the output in a single forward pass, the model incrementally refines its prediction across steps. Final prediction is obtained by weighted aggregation $\hat{y}$ is obtained by summing the contributions from all decision steps (Equation 7):

$$\hat{y}=\gamma_{t}\sum_{t=1}^{T}\;P_{t}$$ (7)

where $\gamma_{t}$ is the step-wise importance weights. The mask is learned through an attention mechanism over the input feature embeddings.

The core training objective of the TabNet model is to minimize the discrepancy between predicted and true oxide contents. Given the multivariate regression setting, the primary loss function employed is the Mean Squared Error (MSE). To further promote sparsity in feature selection, TabNet incorporates a regularization term based on the Kullback-Leibler (KL) divergence between the learned attention masks and a uniform prior distribution. The loss function combines the MSE for regression and a sparsity-inducing regularization term L_sparsity . The total loss is defined as Equation 8:

$$L\_\text{total}=\text{MSE}\left(y,\hat{y}\right)+\lambda^{⋆}L\_\text{sparsity},\text{where }L\_\text{sparsity}=\Sigma_{t}{\left|\left|M^{t}\right|\right|}_{1}$$ (8)

This multi-object loss facilitates shared representation learning while treating each oxide prediction as a separate regression sub-task. This sequential approach allows subsequent steps to focus on correcting the residuals from earlier ones, enabling the model to progressively enhance its estimates. This is particularly advantageous in spectral data, where meaningful signals may be distributed nonlinearly and across non-contiguous wavelengths.

## 3 Results and discussion

To validate the effectiveness of the proposed TabNet method, this paper conducted extensive experiments on two datasets collected from different production regions: Qufu and Linyi. The workflow encompassed datasets, experiments setup, results analysis and discussion.

### 3.1 Dataset

The dataset used in this study was sourced from Liu et al. (2024), which covers cement production sites in Qufu and Linyi, China. The dataset contains NIR spectra and oxide compositions measured using standard analytical methods. To ensure consistent spectral acquisition, measurements were conducted at 24 °C–26 °C, 45%–55% humidity, averaging 64 scans at 4 cm^-1^ resolution to improve signal-to-noise ratio. The spectral data covered a wavenumber range of 3998.14–9998.25 cm^-1^, providing approximately 3,000 wavebands per sample (∼1.93 cm^-1^ spacing), effectively capturing key molecular vibrations associated with Si-O, Al-O, and Fe-O bonds present in cement clinker. The details of both datasets are shown in Table 1. The dataset was randomly divided into training (80%) and testing (20%) subsets to facilitate model evaluation. Our work applies it in a new context by utilizing the TabNet framework for multi-oxide prediction, which is not addressed in Liu et al. (2024). The primary contribution of our study lies in the innovative application of TabNet to this dataset, leading to improved predictive performance and insights into cross-regional variability. The dataset setup also enables a comparative analysis of regional variability in oxide prediction, highlighting differences in raw material composition or manufacturing conditions between Qufu and Linyi. The training data was used to train the model, while the testing data was reserved as a hold-out set for final model evaluation to ensure that no data leakage occurred between the two. The test set represents unseen data that was not involved in model fitting, making it a true hold-out test set for assessing the model’s generalization ability.

**TABLE 1:** Basic information of the datasets samples.

Region	Dataset	Number of samples	Number of features	Spectral band range
Qufu	training	76	3112	3998.14 cm^-1^ ∼ 9998.25 cm^-1^
	testing	20	3112	3998.14 cm^-1^ ∼ 9998.25 cm^-1^
Linyi	training	64	3112	3998.14 cm^-1^ ∼ 9998.25 cm^-1^
	testing	17	3112	3998.14 cm^-1^ ∼ 9998.25 cm^-1^

To reduce noise in the NIR spectral data, this paper applied consistent preprocessing steps across all samples. Specifically, z-score normalization was used to standardize the features, facilitating gradient-based optimization in the TabNet framework. Unlike conventional approaches, the paper deliberately avoided principal component analysis (PCA) or other dimensionality reduction techniques to emphasize TabNet’s intrinsic capability for efficient feature selection and handling of high-dimensional inputs.

### 3.2 Experiment setup and evaluation

The proposed TabNet method was configured with the following hyperparameters. The model was constructed with 5 decision steps and an attention dimensionality of 16, providing adequate capacity for learning relationships in high-dimensional spectral data. For the training protocol, it used a batch size of 256, the Adam optimizer with an initial learning rate of 0.02, and a scheduler for adaptive learning rate adjustment. To ensure stable convergence, the paper implemented a ReduceLROnPlateau scheduler that dynamically adjusted the learning rate by a factor of 0.5 when the validation loss plateaued for 15 consecutive epochs. All experiments were conducted under a fixed random seed (42) to ensure reproducibility. Meanwhile, sparsity regularization was introduced with a coefficient γ = 1.3, enhancing interpretability by promoting sparse attention masks and activation functions. The implementation was built upon PyTorch with the PyTabNet library, with training performed on NVIDIA RTX 3090 GPUs. To evaluate generalization performance and mitigate sampling bias, a multi-fold cross-validation strategy was employed. To rigorously evaluate generalization performance and mitigate potential sampling bias, the paper employed a comprehensive k-fold cross-validation strategy (k = 5) on the training data, while maintaining a strict hold-out test set (20% of total samples) for final performance assessment. This dual-validation approach ensures reliable estimation of the model’s predictive capability on unseen data while providing robust hyperparameter tuning.

To evaluate the performance of method on cement oxide content prediction, the paper adopt three widely recognized regression metrics: Root Mean Square Error (RMSE) (Equation 9), Mean Absolute Error (MAE) (Equation 10), and the Coefficient of Determination (R^2^) (Equation 11). RMSE measures the average magnitude of prediction errors and penalizes larger deviations more heavily due to the squaring operation:

$$R\text{MSE}=\frac{1}{n}\sqrt{\sum_{i=1}^{n}{\left(y_{i}-\hat{y_{i}}\right)}^{2}}$$ (9)

where $y_{i}$ and $\hat{y_{i}}$ denote the ground truth and predicted values, respectively, and n is the total number of samples. Because the errors are squared before they are averaged, RMSE gives relatively high weight to large errors. Lower RMSE indicates better model performance. Therefore, it is particularly sensitive to outliers and is useful in scenarios like cement raw material control.

Compared to RMSE, MAE provides a robust, scale-aware measure of the average absolute error:

$$\text{MAE}=\frac{1}{n}\sum_{i=1}^{n}\left|y_{i}-\hat{y_{i}}\right|$$ (10)

It is more robust to outliers and offers a straightforward interpretation in the same units as the objects variable (e.g., oxide weight percent).

R^2^ quantifies the proportion of variance in the actual values that is captured by the model predictions. Its value range is between −1 and 1. A value of one implies perfect prediction, while 0 indicates the model performs no better than predicting the mean:

$$R^{2}=1-\frac{\sum_{i=1}^{n}{\left(y_{i}-\hat{y_{i}}\right)}^{2}}{\sum_{i=1}^{n}{\left(y_{i}-\hat{y_{i}}\right)}^{2}}$$ (11)

### 3.3 Results analysis

#### 3.3.1 Oxides prediction analysis

The predictive performance for each oxide component is summarized in Table 2. On the Qufu dataset, the TabNet model demonstrated excellent predictive capability, achieving an average RMSE of 0.097, MAE of 0.075, and an average R^2^ of 0.922 across all oxides. Specifically, CaCO_3_ (RMSE = 0.142, MAE = 0.111 and R^2^ = 0.965) and SiO_2_ (RMSE = 0.149, MAE = 0.111, R^2^ = 0.971) exhibited consistently high prediction fidelity. Al_2_O_3_ achieved the lowest RMSE of 0.085 and a strong R^2^ of 0.951, suggesting that its spectral characteristics were effectively captured. Although Fe_2_O_3_ had the lowest RMSE of 0.015, it recorded the lowest R^2^ of 0.802 due to its narrow concentration range. Multi-fold cross-validation further confirmed the robustness of the predictions, with an average MSE cross-validation of 0.080. Among all oxides, Fe_2_O_3_ showed particularly low RMSE values (0.015) due to its limited variability, whereas SiO_2_ and CaCO_3_ exhibited slightly higher RMSE values, reflecting broader compositional ranges. These findings confirm that TabNet effectively captures the complex spectral-chemical relationships in Qufu raw materials, delivering highly accurate and reliable predictions.

**TABLE 2:** Testing performance for Qufu and Linyi datasets.

Region	Oxide	RMSE	MAE	R^2^	MSE cross-val
**Qufu**	CaCO_3_	0.142	0.111	0.965	0.097
	SiO_2_	0.149	0.116	0.971	0.172
	Al_2_O_3_	0.085	0.062	0.951	0.036
	Fe_2_O_3_	0.015	0.012	0.802	0.015
	Avg Oxide	**0.097**	**0.075**	**0.922**	**0.080**
**Linyi**	CaCO_3_	0.153	0.118	0.962	0.100
	SiO_2_	0.154	0.121	0.970	0.180
	Al_2_O_3_	0.088	0.064	0.949	0.040
	Fe_2_O_3_	0.019	0.014	0.793	0.016
	Avg Oxide	**0.104**	**0.079**	**0.919**	**0.084**

The bolded parts in the experimental results indicate the best performance.s

To further evaluate generalization ability, experiments were also conducted on the Linyi dataset. TabNet maintained similarly strong performance, with an average RMSE of 0.104, MAE of 0.079, and R^2^ of 0.919. Both CaCO_3_ (RMSE = 0.153, MAE = 0.118, R^2^ = 0.962) and SiO_2_ (RMSE = 0.154, MAE = 0.121, R^2^ = 0.970) showed comparable accuracy to Qufu, demonstrating the stability of TabNet across different regions. Al_2_O_3_, and Fe_2_O_3_ exhibited slightly higher RMSE values (0.088 and 0.019, respectively) compared to Qufu. This discrepancy is partly due to outliers, which increase prediction variance and lower R^2^. In particular, Fe_2_O_3_ had a lower R^2^ of 0.793 despite small absolute errors, likely due to regional composition differences or spectral noise. The overall predictive performance in Linyi remained strong and closely aligned with the results from Qufu.

**FIGURE 2:**
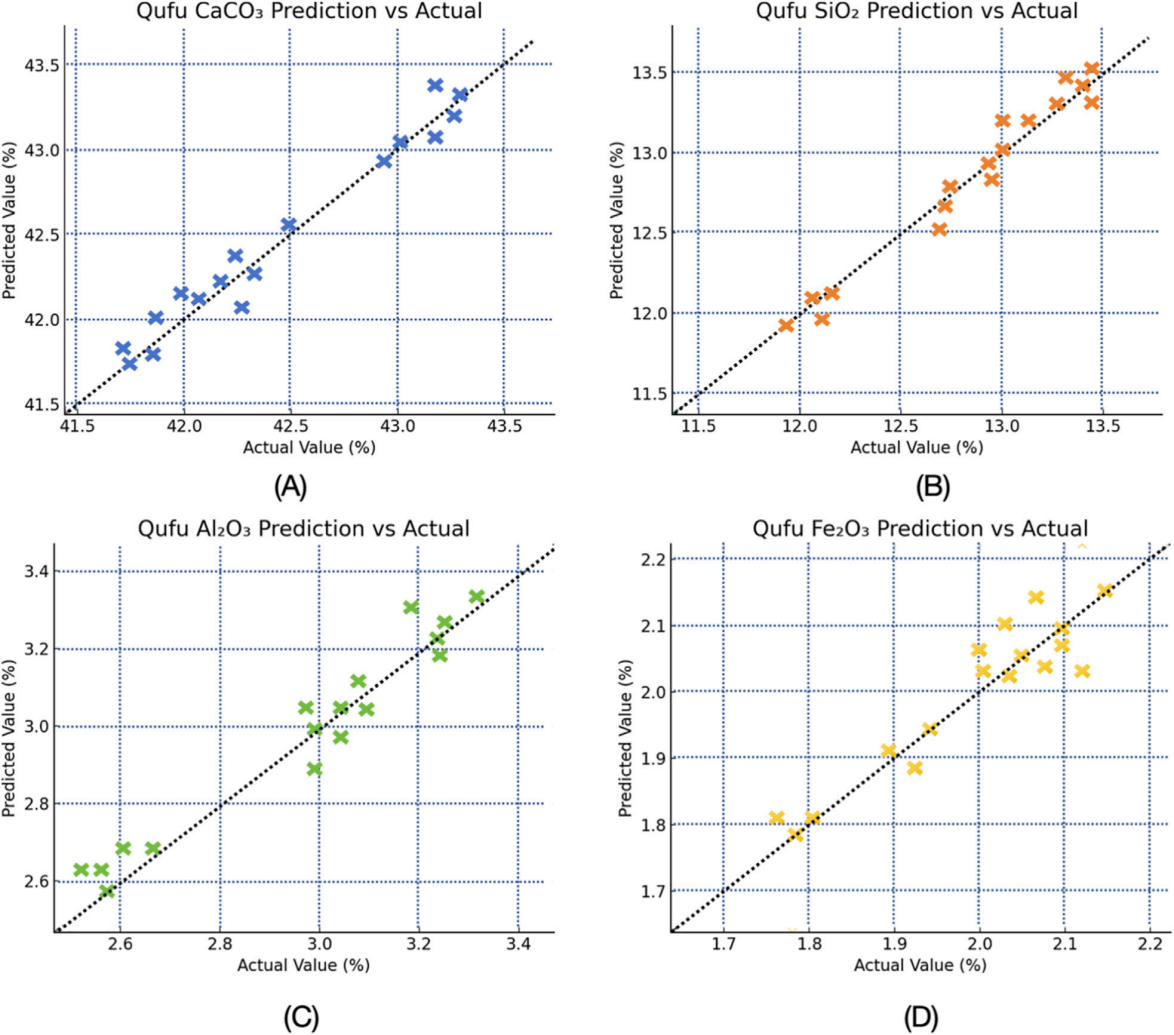
Scatter plots of TabNet predicted *versus* actual oxide concentrations on the test set from Qufu dataset. **(A)** CaCO_3_. **(B)** SiO_2_. **(C)** Al_2_O_3_. **(D)** Fe_2_O_3_.

To provide an intuitive assessment of regression performance, the paper further visualized the relationship between predicted and actual oxide contents using scatter plots. A detailed visualization of the model’s predictive accuracy is presented in Figure 2, which displays the scatter plots of predicted *versus* reference concentrations for the four major oxides using the Qufu dataset. Each data point in the figure represents an individual prediction obtained through our cross-validation procedure, providing a comprehensive assessment of model performance across the entire dataset. Different colors denote different oxides. TabNet demonstrates strong predictive performance, with most points closely aligning along the ideal line and a clear linear correlation between predicted and actual values. For CaCO_3_, predictions align closely with the ground truth, with most errors confined within ±0.15%. This high accuracy is attributed to TabNet’s ability to capture carbonate absorption features in the 4,300–4,500 cm^-1^. Predictions for SiO_2_ also demonstrate strong linearity, with an R^2^ of approximately 0.97. A slight overestimation is observed at lower concentrations, while higher concentrations (∼15%) are nearly perfectly predicted. For Al_2_O_3_, TabNet achieves the lowest prediction error (RMSE = 0.085), with scatter points almost perfectly overlapping the ideal line and showing no systematic deviation. This result highlights the model’s capability to leverage strong Al-O absorption responses in the 4,800–5,500 cm^-1^ region, delivering highly accurate and stable predictions. In the case of Fe_2_O_3_, while the absolute prediction error remains minimal (RMSE = 0.015), the R^2^ value (0.802) is lower than for the other oxides. This discrepancy is primarily due to Fe_2_O_3_’s narrow concentration range, which limits variance explanation despite accurate predictions. Although slight discrepancies were observed—such as higher RMSE for Al_2_O_3_ and Fe_2_O_3_ in Linyi—these variations were attributed to outliers and differences in regional compositions, which could be addressed by model fine-tuning.

#### 3.3.2 Cross-regional generalization analysis

To rigorously evaluate the cross-regional generalizability of the proposed TabNet model, a stringent inter-regional generalization experiment was conducted. This test goes beyond simple hold-out validation and assesses the model’s ability to perform well on data from a completely different production region. We trained the TabNet model on the entire dataset from one region (including both training and testing splits) and then evaluated it directly on the entire, unseen dataset from the other region. This process was performed in both directions: “Train on Qufu, Test on Linyi” and “Train on Linyi, Test on Qufu”. The results of this challenging experiment are summarized in Table 3. As expected, the performance metrics under this cross-regional setting are lower than those obtained from intra-regional testing.

**TABLE 3:** Performance comparison of regression models.

Training region	Testing region	Oxide	RMSE	MAE	R^2^
Qufu	Linyi	CaCO_3_	0.241	0.189	0.901
		SiO_2_	0.263	0.205	0.892
		Al_2_O_3_	0.152	0.118	0.823
		Fe_2_O_3_	0.035	0.027	0.701
		Avg Oxide	**0.173**	**0.135**	**0.829**
Linyi	Qufu	CaCO_3_	0.255	0.201	0.887
		SiO_2_	0.248	0.192	0.905
		Al_2_O_3_	0.161	0.125	0.810
		Fe_2_O_3_	0.031	0.024	0.725
		Avg Oxide	**0.174**	**0.136**	**0.832**

Bold values indicate the average performance.

When trained on Qufu data and tested on Linyi data, TabNet achieved an average R^2^ of 0.829 and RMSE of 0.173 across all oxides. The reverse scenario (Train on Linyi, Test on Qufu) yielded highly consistent results (Average R^2^ = 0.832, RMSE = 0.174), confirming the stability of our method. Notably, the predictions for CaCO_3_ and SiO_2_ remained strong in both directions, with R^2^ values consistently above 0.88. This can be attributed to the well-defined and strong absorption features of carbonate and silicate groups in the NIR spectrum, which the model can reliably identify even amidst regional variations.

The performance for Al_2_O_3_, and Fe_2_O_3_ saw a more noticeable drop (R^2^ between 0.70 and 0.82). This is a foreseeable outcome, as the spectral signatures of these oxides, particularly Fe_2_O_3_, can be more subtle and more susceptible to being influenced by region-specific impurities, particle size distribution, and the complex matrix effects within the raw meal. The model’s ability to maintain reasonable accuracy on entirely unseen data from a different production site underscores its potential for deployment in real-world industrial settings where raw material sources and conditions can vary.

#### 3.3.3 Performance comparison of existing models

To comprehensively assess performance, six regression approaches were compared, including Partial Least Squares Regression (PLSR), Random Forest (Statistics and Breiman, 2001), Linear Regression (Maulud and Abdulazeez, 2020), Wavelength selection CARS (Zhang Y et al., 2024), SPORT (Zhang et al., 2023) and the proposed TabNet method. To ensure a comprehensive evaluation, we expanded our benchmark suite by incorporating two additional nonlinear models: Support Vector Machine (SVM) (Meza Ramire et al., 2020) and a Feedforward Artificial Neural Network (ANN) (Zhang Y et al., 2024). The ANN comprised two hidden layers with 64 and 32 neurons, respectively, and used ReLU activation, trained with the Adam optimizer. The SVM model was configured with a carefully tuned regularization parameter and kernel coefficient. The results are summarized in Table 4.

**TABLE 4:** Performance comparison of regression models.

Oxide	Method	RMSE	R^2^	MSE cross-val
CaCO_3_	PLSR	0.230	0.910	0.150
	Random Forest	0.204	0.941	0.125
	Linear Regression	0.225	0.923	0.140
	Selection CARS	0.182	0.9489	0.120
	SPORT	0.217	0.925	0.137
	SVM	0.188	0.943	0.129
	ANN	0.175	0.951	0.115
	**TabNet**	**0.142**	**0.965**	**0.097**
SiO_2_	PLSR	0.220	0.920	0.240
	Random Forest	0.190	0.940	0.207
	Linear Regression	0.210	0.925	0.220
	Selection CARS	0.180	0.932	0.190
	SPORT	0.208	0.933	0.224
	SVM	0.195	0.935	0.201
	ANN	0.169	0.949	0.178
	**TabNet**	**0.149**	**0.971**	**0.172**
Al_2_O_3_	PLSR	0.135	0.900	0.070
	Random Forest	0.110	0.925	0.055
	Linear Regression	0.130	0.910	0.065
	Selection CARS	0.100	0.931	0.045
	SPORT	0.128	0.912	0.061
	SVM	0.115	0.922	0.058
	ANN	0.098	0.938	0.049
	**TabNet**	**0.085**	**0.951**	**0.036**
Fe_2_O_3_	PLSR	0.045	0.720	0.025
	Random Forest	0.030	0.750	0.020
	Linear Regression	0.042	0.735	0.023
	Wavelength selection CARS	0.023	0.765	0.019
	SPORT	0.040	0.743	0.021
	SVM	0.028	0.758	0.022
	ANN	0.021	0.781	0.018
	**TabNet**	**0.015**	**0.802**	**0.015**

Bold values indicate the best performance.

Traditional linear models such as PLSR and Linear Regression demonstrated the highest prediction errors across all oxides. To ensure a fair comparison, we specify the type of data used for each regression model. They were applied to the full-spectrum data containing all 3,112 spectral features. On the other hand, Selection CARS and SPORT models utilized pre-selected wavelength subsets, where a smaller set of informative wavelengths was chosen using feature selection techniques prior to training. The TabNet model was also trained on full-spectrum data. For instance, CaCO_3_ exhibited RMSE values of 0.230 and 0.225 for PLSR and LR, respectively. While PLSR reduces collinearity by projecting features into latent variables, its linear nature limits its ability to capture subtle nonlinear patterns, resulting in suboptimal R^2^ scores. Random Forest improved predictive accuracy compared to PLSR and LR, as it can handle nonlinear interactions among spectral features. For example, the RMSE for Al_2_O_3_, decreased to 0.110 compared to 0.135 for PLSR. Nevertheless, Random Forest struggled to achieve high variance explanation for Fe_2_O_3_ (R^2^ = 0.750).

The newly introduced SVM and ANN models demonstrated competitive performance, solidly outperforming the linear models and aligning more closely with Random Forest. Notably, the ANN emerged as a particularly strong baseline, achieving the second-best performance for several oxides (e.g., CaCO_3_ and Al_2_O_3_). The prediction of CaCO_3_ achieved notably performance with an RMSE of 0.175, R^2^ of 0.951, and cross-validated MSE of 0.115, underscoring the capability of nonlinear function in capturing the complex spectral-carbonate relationships. This comprehensive comparison reinforces that TabNet’s success is not merely due to its non-linear nature, but to its sophisticated feature selection and sequential attention mechanism, which allows it to more effectively model the complex spectral-oxide relationships in an end-to-end manner.

Feature selection approaches such as CARS and SPORT reduced feature redundancy by identifying informative wavelengths. This strategy substantially improved prediction performance compared to simple linear methods. For instance, SPORT achieved an RMSE of 0.128 for Al_2_O_3_, *versus* 0.130 for Linear Regression. However, both methods rely on manual wavelength engineering, making them sensitive to noise and less effective in capturing nonlinear relationships. In contrast, the proposed TabNet model consistently delivered the best predictive performance across all oxides. TabNet achieved the lowest cross-validation errors among all evaluated oxides, with an average MSE of approximately 0.08, significantly outperforming feature selection methods CARS and SPORT, which yielded MSE values ranging from 0.12 to 0.22. Unlike traditional methods, TabNet eliminates the need for manual feature selection or complex preprocessing by integrating end-to-end learning, sparse attention, and nonlinear decision steps.

**FIGURE 3:**
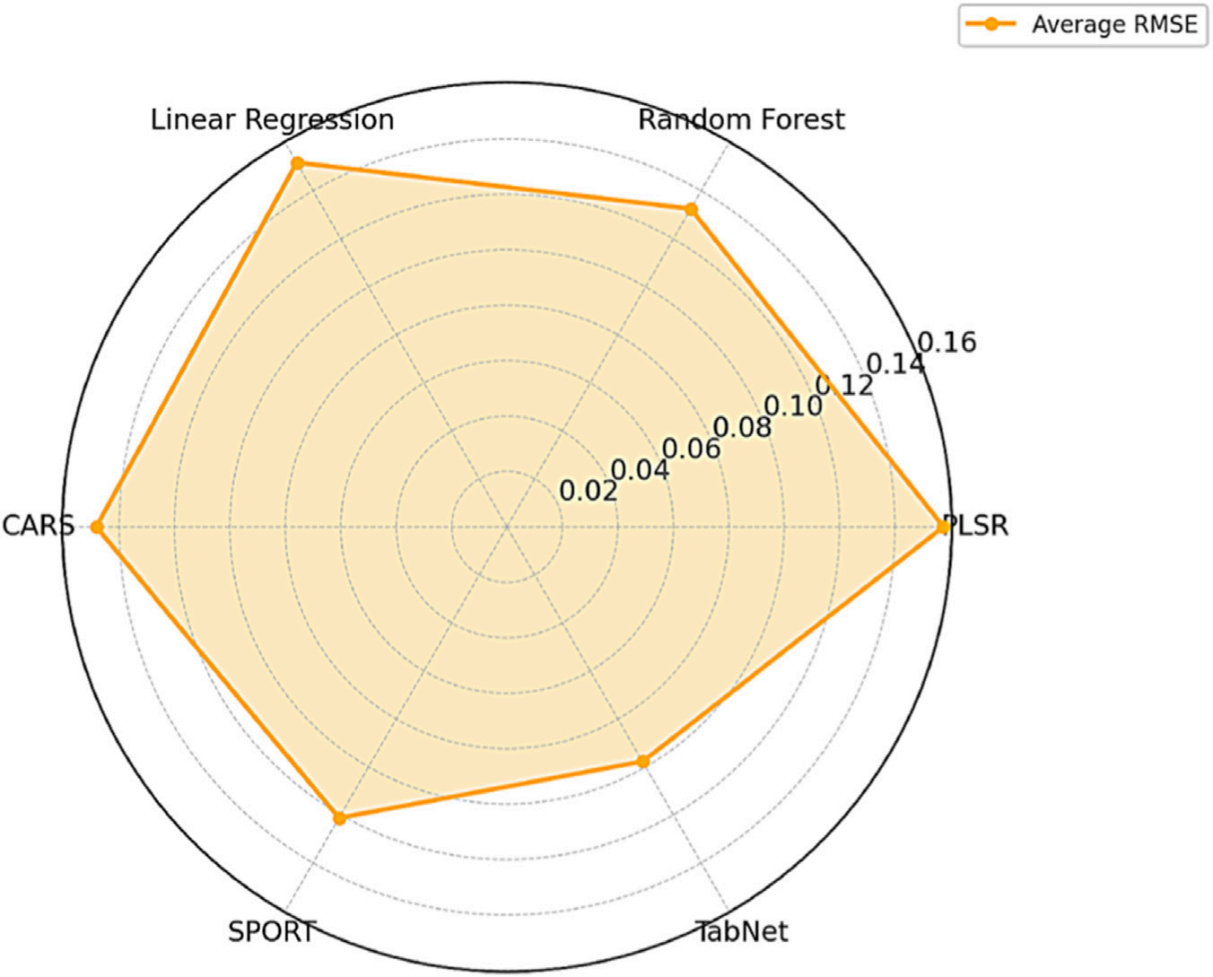
Average RMSE radar chart for models.

In addition, we present an intuitive comparison of model performance. As shown in Figure 3, the radar chart illustrates the average RMSE of each regression model across all oxides, where a smaller enclosed area represents lower prediction error and better overall performance. Notably, TabNet forms the smallest and most compact polygon, reflecting both the lowest average RMSE and the most balanced prediction across oxide types. These visual results align closely with the tabular analysis, further confirming TabNet’s ability to automatically select informative spectral bands and capture nonlinear dependencies via its sparse attention mechanism. It is a robust and scalable solution for multi-oxide prediction in cement quality monitoring.

#### 3.3.4 Discussion

To complement the quantitative results, a qualitative analysis was conducted to explore the interpretability of TabNet in predicting oxide compositions. TabNet’s sparse attention mechanism dynamically adjusts feature selection across decision steps, allowing the model to focus on the most relevant spectral regions for each oxide. The feature importance identified by TabNet provides a direct, data-driven window into the underlying chemistry of the cement raw meal. As shown in Table 5, the top-10 most relevant wavebands selected for CaCO_3_, SiO_2_, Al_2_O_3_, and Fe_2_O_3_ are primarily distributed within the 4,200–9500 cm^-1^ range. This aligns well with known short-wave infrared (SWIR) absorption bands commonly observed in cementitious materials, confirming the chemical plausibility of the selected features. The high relevance of specific wavelengths, as listed in Table 5, is not coincidental but aligns with fundamental molecular vibrations. A paramount example is the dominant role of the ∼4,300 cm^-1^ band for predicting CaCO_3_ content. This band is mechanistically assigned to the strong combination band of the carbonate ion (CO_3_^2-^), which serves as the primary spectral fingerprint for calcite. The model’s autonomous and emphatic selection of this band provides a compelling validation of its chemical relevance and confirms that it is learning meaningful patterns.

**TABLE 5:** The top-10 most relevant wavebands selected for oxides.

Oxide	Top 10 wavelength (cm^-1^)
CaCO_3_	4,300, 4,800, 5,200, 5,400, 5,700, 6,000, 6,500, 7,200, 7,600, 8,700
SiO_2_	4,500, 4,800, 5,200, 6,000, 6,900, 7,200, 7,600, 8,200, 8,700, 9500
Al_2_O_3_	4,500, 5,200, 5,700, 6,000, 6,800, 7,100, 7,200, 7,600, 8,200, 8,900
Fe_2_O_3_	4,200, 4,500, 4,800, 5,200, 6,000, 6,500, 7,200, 7,600, 8,700, 9500

Specifically, CaCO_3_ shows unique peaks at 4,300 and 5,400 cm^-1^linked to carbonate stretching vibrations; SiO_2_ has distinctive high-wavelength peaks at 6,900, 8,200, and 9500 cm^-1^ associated with silicate network vibrations; Al_2_O_3_ features absorption at 7,100 and 8,900 cm^-1^ due to hydroxyl-alumina bonds; while Fe_2_O_3_ displays low-to mid-range peaks at 4,200 and 6,500 cm^-1^ corresponding to crystal field transitions of iron oxides. Importantly, TabNet successfully implemented an automatic feature selection mechanism, capturing chemically relevant spectral regions without manual engineering. Feature analysis further revealed that both regions shared nearly identical top spectral bands, with only the 10th most important band differing (6,900.63 cm^-1^ in Qufu vs. 6,750.63 cm^-1^ in Linyi). This subtle spectral shift may partially explain the small performance gap observed for Fe_2_O_3_. These results suggested that region-aware model fine-tuning could further improve cross-regional generalization and robustness.

**FIGURE 4:**
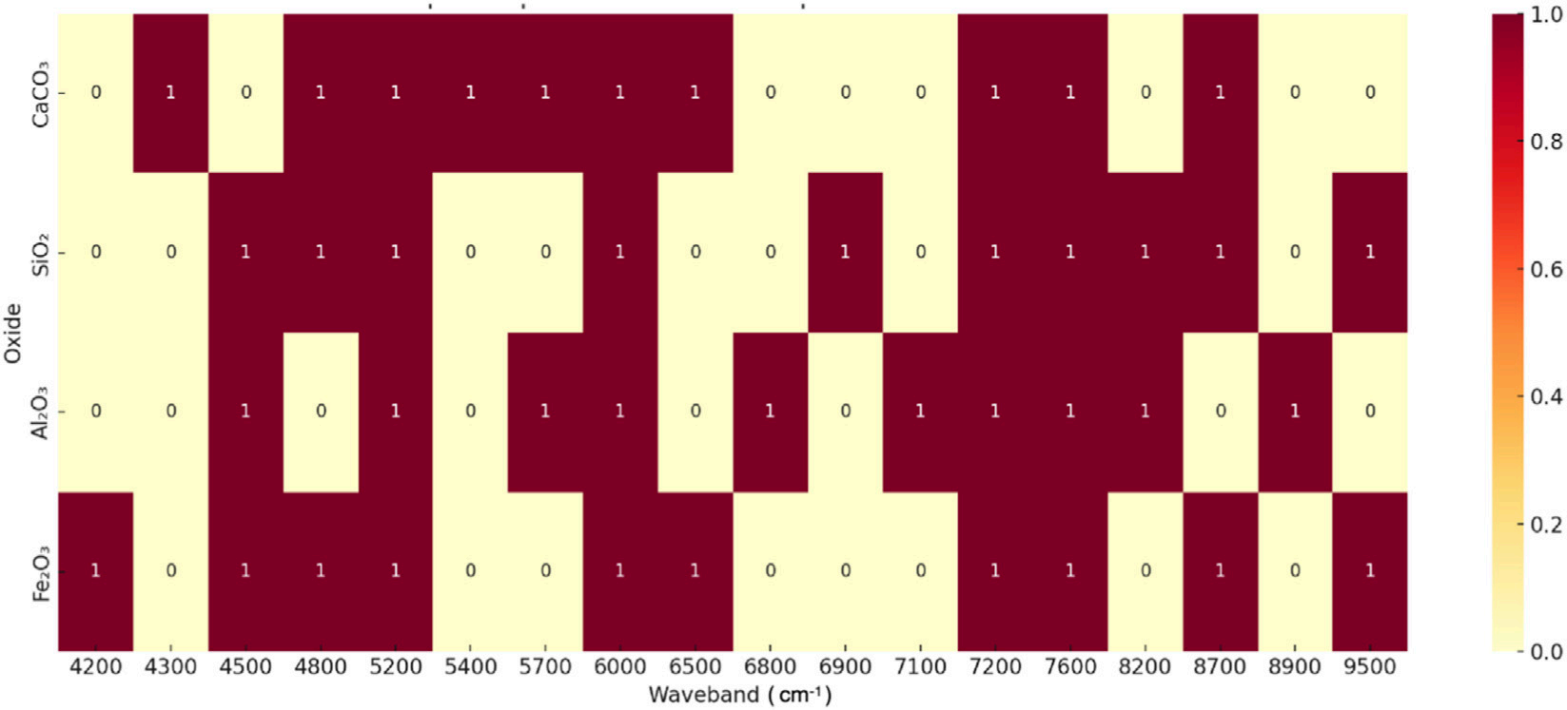
Heatmap of Top 10 Wavebands importance score for four Oxides.

The feature importance heatmap is shown in Figure 4, illustrating substantial overlap among the top-10 wavebands across the four oxides. Notably, wavebands at 5,200, 6,000, 7,200, 7,600, and 8,700 cm^-1^ correspond to fundamental molecular overtones and combination vibrations common to carbonate, hydroxyl, and silicate groups. Despite these shared features, the model effectively differentiates between oxides by leveraging distinctive wavebands: CaCO_3_ at 4,300 cm^-1^, Al_2_O_3_ at 7,100 cm^-1^, and Fe_2_O_3_ at 4,200 cm^-1^. The heatmap also highlights stronger importance of high-wavelength regions (9500 cm^-1^) for Fe_2_O_3_ and SiO_2_, and unique absorption features for Al_2_O_3_ in the 7,100–8,900 cm^-1^ range, whereas CaCO_3_ relies primarily on low-to-mid wavelength regions (4,300–5,400 cm^-1^) for accurate prediction. Peaks near 7,200 and 5,200 cm^-1^ are predominantly attributed to water and alumina-related vibrations, the strong absorption at 4,300 cm^-1^ originates from carbonate symmetric stretching, and weaker absorption near 4,500 cm^-1^ likely results from overlapping contributions of Al_2_O_3,_ Fe_2_O_3_ and SiO_2_. This connection transcends mere spectral assignment and has direct implications for predicting material properties. In cement manufacturing, the precise content of CaCO_3_ is critical as it governs the burnability of the raw meal and the thermodynamics of clinker phase formation. Therefore, the model’s accurate identification of the ∼4,300 cm^-1^ band is not just a statistical outcome. It is the foundational step in a chain of reasoning that links a spectral feature to a core material performance property.

The study note that the relatively small sample size, particularly for the Linyi dataset, may limit the statistical robustness and generalization of our model. While the current results demonstrate strong predictive performance, larger datasets would allow for more comprehensive evaluation, especially in capturing less common spectral variations and regional differences. In future work, we plan to collect larger-scale datasets from multiple production regions, which will not only improve cross-regional generalization but also further validate the applicability and scalability of the proposed TabNet framework for industrial cement quality monitoring.

## 4 Conclusion

The paper proposed a TabNet-based multi-output regression framework for accurate and interpretable prediction of key oxide contents in cement raw meals using NIR spectroscopy. Unlike traditional chemometric models, TabNet leverages sparse attention and sequential feature selection to automatically identify chemically meaningful spectral bands, enabling robust modeling of the complex nonlinear relationships inherent in high-dimensional spectral data. Extensive experiments on two real-world datasets from Qufu and Linyi demonstrated that the proposed method consistently outperforms baseline models such as PLSR and Random Forest in terms of both RMSE and R^2^. In addition to its superior predictive accuracy, the model offers interpretability through visualizable attention masks, which align with known oxide-specific SWIR absorption bands. Overall, this work presents a scalable, end-to-end solution for real-time cement quality monitoring. The proposed framework extends naturally to other spectral-based tasks, including inorganic material analysis, geochemical sensing, and process monitoring.

## Data Availability

The raw data supporting the conclusions of this article will be made available by the authors, without undue reservation.
